# Atrial fibrillation and implantable cardioverter-defibrillator in non-ischaemic heart failure with reduced ejection fraction: insights from the DANISH trial

**DOI:** 10.1093/europace/euaf200

**Published:** 2025-09-24

**Authors:** Seiko N Doi, Adelina Yafasova, Jens Jakob Thune, Jens C Nielsen, Niels E Bruun, Lars Videbæk, Hans Eiskjær, Christian Hassager, Jesper H Svendsen, Dan E Høfsten, Steen Pehrson, Lars Køber, Jawad H Butt

**Affiliations:** Department of Cardiology, Copenhagen University Hospital—Rigshospitalet, Blegdamsvej 9, Copenhagen 2100, Denmark; Department of Preventive Medicine and Epidemiology, National Cerebral and Cardiovascular Center, Suita, Japan; Department of Cardiology, Copenhagen University Hospital—Rigshospitalet, Blegdamsvej 9, Copenhagen 2100, Denmark; Department of Clinical Medicine, University of Copenhagen, Copenhagen, Denmark; Department of Cardiology, Copenhagen University Hospital—Bispebjerg and Frederiksberg, Copenhagen, Denmark; Department of Cardiology, Aarhus University Hospital, Aarhus, Denmark; Department of Clinical Medicine, Aarhus University, Aarhus, Denmark; Department of Clinical Medicine, University of Copenhagen, Copenhagen, Denmark; Department of Cardiology, Zealand University Hospital, Roskilde, Denmark; Department of Cardiology, Aalborg University Hospital, Aalborg, Denmark; Department of Cardiology, Odense University Hospital, Svendborg, Denmark; Department of Cardiology, Aarhus University Hospital, Aarhus, Denmark; Department of Cardiology, Copenhagen University Hospital—Rigshospitalet, Blegdamsvej 9, Copenhagen 2100, Denmark; Department of Clinical Medicine, University of Copenhagen, Copenhagen, Denmark; Department of Cardiology, Copenhagen University Hospital—Rigshospitalet, Blegdamsvej 9, Copenhagen 2100, Denmark; Department of Clinical Medicine, University of Copenhagen, Copenhagen, Denmark; Department of Cardiology, Copenhagen University Hospital—Rigshospitalet, Blegdamsvej 9, Copenhagen 2100, Denmark; Department of Clinical Medicine, University of Copenhagen, Copenhagen, Denmark; Department of Cardiology, Copenhagen University Hospital—Rigshospitalet, Blegdamsvej 9, Copenhagen 2100, Denmark; Department of Cardiology, Copenhagen University Hospital—Rigshospitalet, Blegdamsvej 9, Copenhagen 2100, Denmark; Department of Clinical Medicine, University of Copenhagen, Copenhagen, Denmark; Department of Cardiology, Copenhagen University Hospital—Rigshospitalet, Blegdamsvej 9, Copenhagen 2100, Denmark; Department of Cardiology, Zealand University Hospital, Roskilde, Denmark; Department of Cardiology, Copenhagen University Hospital—Herlev and Gentofte, Hellerup, Denmark

**Keywords:** Heart failure, Implantable cardioverter-defibrillator, Atrial fibrillation, Clinical trial

## Abstract

**Aims:**

Atrial fibrillation (AF) is associated with an increased risk of sudden cardiac death. Therefore, the effect of an implantable cardioverter-defibrillator (ICD) may be greater in patients with AF. We examined the long-term effects of primary prevention ICD implantation vs. usual clinical care according to AF status in DANISH.

**Methods and results:**

Outcomes were analysed according to AF status at baseline (history and/or on enrollment ECG). The primary outcome was all-cause death, and secondary outcomes were cardiovascular and sudden cardiovascular death. Of the 1116 patients with non-ischaemic heart failure with reduced ejection fraction randomized in DANISH, 418 (37.5%) had AF at baseline, of whom 24.2% had paroxysmal AF, 17.0% persistent AF, and 58.9% permanent AF. AF status did not significantly modify the effect of ICD implantation on all-cause death, although there was a suggestion of a greater effect in patients with [hazard ratio (HR) 0.78 (95% CI, 0.59–1.03)] vs. without AF [HR 0.98 (0.75–1.27)] (*P*_interaction_ = 0.15). AF status significantly modified the effect of ICD implantation on cardiovascular death, such that ICD implantation was associated with a lower rate of this outcome in patients with AF [HR 0.67 (0.48–0.94)], but not in those without AF [HR 1.04 (0.76–1.41)] (*P*_interaction_ = 0.04). Although AF status did not significantly modify the effect of ICD implantation on sudden cardiovascular death, there was a suggestion of a greater effect in patients with [HR 0.45 (0.24–0.82)] vs. without AF [HR 0.76 (0.41–1.38)] (*P*_interaction_ = 0.20).

**Conclusion:**

In the DANISH trial, the presence of AF was associated with a greater effect of ICD implantation on cardiovascular death, and although similar trends were observed for all-cause and sudden cardiovascular death, the treatment-by-subgroup interaction was not statistically significant for these outcomes.

**Registration:**

URL: https://www.clinicaltrials.gov; unique identifier: NCT00542945.

## Graphical Abstract

**Graphical Abstract:**
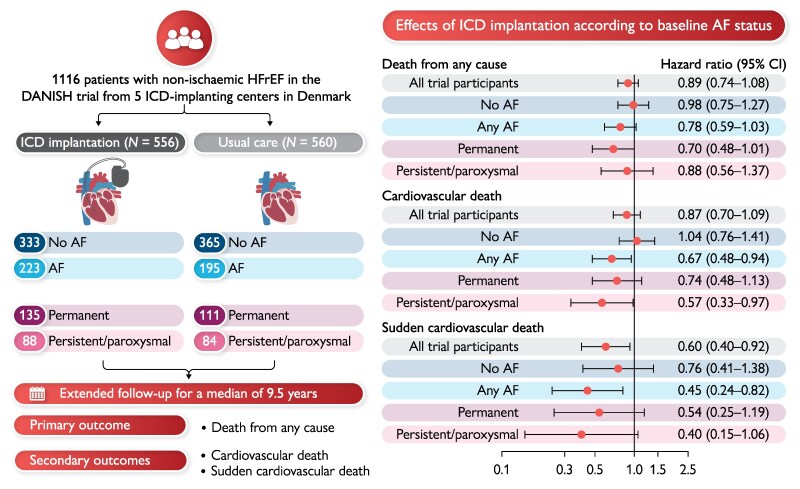
Study flow chart and effect of ICD implantation compared with usual care according to baseline AF status. All hazard ratios are stratified according to centre and cardiac resynchronization therapy implantation (preexisting or planned). AF, atrial fibrillation; CI, confidence interval; DANISH, Danish Study to Assess the Efficacy of Primary Prevention of Implantable Cardioverter Defibrillators in Patients with Non-ischaemic Systolic Heart failure on Mortality; HFrEF, heart failure with reduced ejection fraction; ICD, implantable cardioverter-defibrillator.

What’s new?

- In an extended follow-up analysis of DANISH, AF modified the effect of ICD implantation, such that ICD implantation, compared with usual care, significantly reduced the rate of cardiovascular death in patients with AF, but not in those without AF. A similar non-significant trend was observed for all-cause death and sudden cardiovascular death.
- These findings suggest that individuals with non-ischaemic HFrEF with AF may derive greater benefit from ICDs than those without AF. However, this question can only be answered definitively in a trial specifically designed and powered to address this question.

## Introduction

Atrial fibrillation (AF) is common in heart failure (HF) with reduced ejection fraction (HFrEF), and it is associated with greater morbidity and mortality in these patients.^1,2^ There is also some evidence to suggest that the presence of AF in patients with HFrEF is associated with a higher risk of sudden cardiovascular death, a mode of death which can potentially be prevented by an implantable cardioverter-defibrillator (ICD) device.^3–5^ Thus, it is possible that patients with AF may derive a greater benefit from a primary prevention ICD implantation compared to those without. Conversely, patients with AF are generally older and have a greater comorbidity burden than those without, and despite having a higher risk of sudden cardiovascular death, patients with AF may derive a lesser benefit from ICD implantation.^6–9^ However, data on the effect of ICD implantation according to AF status from subgroup analyses of landmark primary prevention ICD trials have been inconsistent, and a formal interaction test between AF status and treatment effect was either not performed or reported.^10–12^ Importantly, none of these analyses accounted for the type of AF [i.e. whether patients had permanent or non-permanent (paroxysmal or persistent) AF], and this distinction may be important since patients with permanent AF differ substantially from those with non-permanent AF in terms of clinical characteristics and possibly the risk of clinical outcomes.^13,14^

Consequently, we examined these questions in the DANISH Study to Assess the Efficacy of ICDs in Patients with Non-ischaemic Systolic Heart Failure on Mortality (DANISH), which is the most contemporary primary prevention ICD trial in patients with HFrEF.^15^ Specifically, we investigated the long-term efficacy of primary prophylactic ICD implantation in patients with non-ischaemic HFrEF according to AF status at baseline, including type of AF (i.e. permanent vs. non-permanent AF). We also addressed the long-standing question of whether AF is an independent predictor of adverse outcomes or simply a marker of more advanced HF.

## Methods

The DANISH trial was a randomized, controlled, open-label multicentre trial, which evaluated the efficacy and safety of primary prevention ICD implantation compared with usual care in patients with non-ischaemic HFrEF. The design and main findings of the trial have been published and described in detail previously.^15,16^ In brief, 1116 patients, enrolled from five ICD-implanting centres in Denmark between 7 February 2008 and 30 June 2014, were randomized in a 1:1 ratio to ICD implantation or usual care. The protocol was approved by the ethics committee in the Capital Region of Denmark (H-D-2007-0101), and all participants gave written informed consent. The corresponding author had full access to all the trial data and takes responsibility for its integrity and the data analysis.

### Study participants

Patients with a diagnosis of HF were eligible if they had a non-ischaemic cause of HF (preferably determined by coronary angiography, but a normal computed tomographic angiogram or nuclear myocardial perfusion imaging was accepted), had a left ventricular ejection fraction (LVEF) of ≤35%, were in New York Heart Association (NYHA) functional Class II or III [or Class IV if cardiac resynchronization therapy (CRT) was planned], had a N-terminal pro-B-type natriuretic peptide (NT-proBNP) concentration >200 pg/mL, and were optimally treated with medical therapy for HF. Patients with preexisting pacemakers or CRT-pacemakers could also be included in the study if they were willing to accept a potential upgrade. Patients fulfilling indications for a CRT device received a CRT-defibrillator (if randomized to the ICD arm) or a CRT-pacemaker (if randomized to the control arm). Key exclusion criteria were a resting heart rate >100 beats per minute in patients with permanent AF and renal failure that was being treated with dialysis. A full list of exclusion criteria is provided in the main paper.^15^

### Definition of atrial fibrillation

Data on a history (and chronicity) of AF were collected from the trial case report forms (CRF). Investigators were first asked whether patients had a history of AF any time before enrollment and then to specify the type of AF according to the following options: paroxysmal, persistent, and permanent. Data on heart rhythm on the electrocardiogram (ECG) at enrollment were also collected on the CRF, and investigators were asked to specify the heart rhythm from the following options: sinus rhythm, AF, atrial flutter, paced rhythm, and other.

As in previous studies, the definition of AF in the present study included AF or atrial flutter.^17,18^ The assessment of the effect of ICD implantation according to permanent AF status at baseline was a prespecified subgroup analysis. In the present analysis, we made the following comparisons based on AF status at baseline: (i) patients without any AF (no history of AF and no AF on enrollment ECG) vs. any AF (a history of AF or AF on enrollment ECG); (ii) patients without any AF (no history of AF and no AF on enrollment ECG) vs. non-permanent AF (a history of paroxysmal/persistent AF or AF on enrollment ECG without a history of AF) vs. permanent AF (a history of permanent AF). This classification was used in all analyses.

### Follow-up and outcomes

In the main trial, patients were followed from randomization until death or 30 June 2016, whichever came first. In the present study with extended follow-up, patients were followed until death or 18 May 2020, whichever came first, and no patients were lost to follow-up.^19^ As in the main trial, the primary outcome was all-cause death, and secondary outcomes were cardiovascular death and sudden cardiovascular death. All outcomes were assessed by review of patients’ electronic and hard copy medical files and adjudicated by an event committee blinded to treatment allocation.

Device complications and shock therapy among patients randomized to an ICD were also examined, although these data were available only for the original, and not the extended, follow-up duration.

### Statistical analyses

Baseline characteristics were summarized as frequencies with percentages, means with standard deviations, or medians with 25th–75th percentiles, and differences were tested using the *χ*^2^ test or the Fisher’s exact test for categorical variables and unpaired *t*-test, the Wilcoxon test, ANOVA, or the Kruskal–Wallis test for continuous variables, as appropriate.

The association between AF status at baseline and outcomes was evaluated using Cox proportional hazards regression models, adjusted for age, sex, treatment assignment, centre, CRT (preexisting or planned), body mass index (BMI), duration of HF, NYHA functional class, log of NT-proBNP, LVEF, estimated glomerular filtration rate (eGFR), history of HF hospitalization, hypertension, and diabetes.

The effect of ICD implantation vs. usual care according to AF status at baseline was examined with the Kaplan–Meier estimator, Aalen–Johansen estimator, and Cox proportional hazards regression models, stratified according to centre and CRT implantation (preexisting or planned). Since there was a difference in sex between the ICD and control arm in patients with non-permanent AF, these analyses were also adjusted for sex.

The effect of ICD implantation was also examined according to continuous age in patients with and without AF at baseline, respectively, using fractional polynomial models, restricted cubic spline models, or linear regression models, whichever had the lowest Akaike information criterion score. These analyses were conducted, because the main DANISH trial demonstrated that ICDs reduced the rate of all-cause death in younger, but not in older, patients.

Data were analysed according to the intention-to-treat principle. All analyses were conducted using STATA version 17.0 and SAS version 9.4. A *P*-value of 0.05 was considered statistically significant.

## Results

Of the 1116 patients randomized in the DANISH trial, 418 (37.5%) had AF at baseline (416 patients with a history of AF; 2 patients with AF on enrollment ECG and no history of AF). Among patients with AF, 246 (58.9%) had permanent AF, 71 (17.0%) had persistent AF, and 101 (24.2%) had paroxysmal AF.

### Patient characteristics

#### Any atrial fibrillation at baseline

Patient characteristics according to AF status at baseline are presented in *Table 1*. Compared with patients without AF, those with AF were older, more often male, and more likely to have a prior stroke, and they had a higher BMI, lower eGFR, and shorter QRS duration. Although patients with AF had a higher LVEF, they had a higher NT-proBNP level and longer duration of HF and were more likely to have a prior HF hospitalization compared with patients without AF. Regarding background HF therapy, there were no significant differences between the groups with respect to treatment with an angiotensin-converting enzyme inhibitor (ACEi)/ARB, beta-blocker, or mineralocorticoid receptor antagonist. However, compared with patients without AF, those with AF were more frequently treated with amiodarone, anticoagulants, and loop diuretics, and they less often had a preexisting or planned CRT implantation.

**Table 1:** Baseline characteristics of the study population according to AF status at baseline

	No AF	AF	*P*-value
	*n* = 698	*n* = 418	
Age, median (interquartile range)	62 (54–69)	66 (60–72)	<0.001
Male sex, *n* (%)	461 (66.1)	348 (83.3)	<0.001
Physiologic measures, median (interquartile range)			
Systolic blood pressure, mmHg	125 (110–140)	122 (110–136)	0.07
Heart rate, b.p.m.	68 (61–77)	69 (60–78)	0.37
BMI, kg/m^2^	26 (23–30)	28 (25–31)	<0.001
NT-proBNP, pg/mL	990 (500–2095)	1476 (755–2689)	<0.001
eGFR, mL/min/1.73 cm^2^	77 (62–96)	65 (53–82)	<0.001
QRS duration, ms	149 (116–166)	138 (105–164)	<0.001
LVEF, %, mean (SD)	23.8 (6.4)	24.6 (5.9)	0.02
Duration of HF, median (interquartile range), months	13 (7–52)	36 (12–86)	<0.001
Main cause of HF, *n* (%)			0.17
Idiopathic	529 (75.8)	320 (76.6)	
Valvular	20 (2.9)	21 (5.0)	
Hypertension	80 (11.5)	37 (8.9)	
Other	69 (9.9)	40 (9.6)	
NYHA class, *n* (%)			0.19
II	384 (55.0)	213 (51.0)	
III/IV	314 (45.0)	205 (49.0)	
Medical history, *n* (%)			
Hospitalization for HF	432 (62.3)	289 (69.6)	0.01
Hypertension	209 (29.9)	139 (33.3)	0.24
Diabetes	127 (18.2)	84 (20.1)	0.43
Stroke	58 (8.3)	58 (13.9)	0.003
COPD	82 (11.8)	53 (13.0)	0.57
Treatment, *n* (%)			
ACEI/ARB	675 (96.7)	402 (96.2)	0.64
Beta-blocker	643 (92.1)	383 (91.6)	0.77
MRA	411 (58.9)	235 (56.2)	0.38
Amiodarone	12 (1.7)	54 (12.9)	<0.001
Loop diuretic	498 (71.4)	335 (80.1)	<0.001
Antiplatelet	340 (48.7)	121 (29.0)	<0.001
Anticoagulant	89 (12.8)	337 (80.6)	<0.001
Preexisting or planned CRT	430 (61.6)	215 (51.4)	<0.001

ACEI, angiotensin-converting enzyme inhibitor; AF, atrial fibrillation; ARB, angiotensin receptor blocker; BMI, body mass index; COPD, chronic obstructive pulmonary disease; CRT, cardiac resynchronization therapy; eGFR, estimated glomerular filtration rate; HF, heart failure; LVEF, left ventricular ejection fraction; MRA, mineralocorticoid receptor antagonist; NYHA, New York Heart Association; NT-proBNP, N-terminal pro-B-type natriuretic peptide.

Patient characteristics according to treatment assignment and AF status at baseline are shown in Supplementary material online, *Table S1*. Overall, patient characteristics were similar between the ICD and usual care groups in both patients with and without AF.

### Type of atrial fibrillation at baseline

Patient characteristics according to type of AF at baseline (i.e. no AF vs. non-permanent AF vs. permanent AF) are presented in Supplementary material online, *Table S2*. Patients with permanent AF were older, more often male, and had a lower eGFR and a higher NT-proBNP level. They were also more likely to have a prior stroke and a longer duration of HF. Regarding background HF therapy, there was no significant difference between the groups. The proportion of patients treated with amiodarone was highest in the non-permanent AF group, whereas the proportion of patients treated with anticoagulants was the highest in the permanent AF group. The proportion of patients treated with loop diuretics was highest in patients with AF, with no difference between the non-permanent and permanent AF groups. Patients with permanent AF less often had a preexisting or planned CRT implantation.

When baseline characteristics were examined according to treatment assignment and type of AF at baseline, there was no significant differences between the usual care arm and the ICD arm across AF types, with one exception: among participants with non-permanent AF, the proportion of males was significantly lower in the usual care arm than in the ICD arm (see Supplementary material online, *Table S3*).

### Outcomes according to atrial fibrillation status at baseline

#### Any atrial fibrillation at baseline

The median follow-up was 9.5 years (25th–75th percentile 7.9–10.9 years). The absolute risk and rate of outcomes according to LVEF are shown in *Table 2* and Supplementary material online, *Figure S1*. In unadjusted analyses, AF at baseline was associated with a significantly higher rate of all the outcomes examined. After adjustment for prognostic variables (excluding NT-proBNP), these associations were generally attenuated; AF at baseline was associated with a significantly higher rate of cardiovascular [hazard ratio (HR) 1.28 (1.01–1.62)] and sudden cardiovascular death [HR 1.55 (1.00–2.42)], but not all-cause death, although there was a trend towards a higher rate of all-cause death with AF [HR 1.21 (0.99–1.47)]. After further adjustment for NT-proBNP, AF at baseline was no longer significantly associated with any of the outcomes, but there was a trend towards a higher rate of sudden cardiovascular death with AF [HR 1.49 (0.94–2.34)].

**Table 2:** Outcomes according to AF status at baseline

	No AF	AF
	*n* = 698	*n* = 418
All-cause death		
*n* (%)	232 (33.2)	202 (48.3)
Event rate per 100 person-years (95% CI)	4.3 (3.8–4.9)	6.9 (6.0–7.9)
HR (95% CI)	Reference	1.64 (1.36–1.98)
HR (95% CI)^a^	Reference	1.21 (0.99–1.47)
HR (95% CI)^b^	Reference	1.06 (0.86–1.31)
Cardiovascular death		
*n* (%)	163 (23.4)	148 (35.4)
Event rate per 100 person-years (95% CI)	3.0 (2.6–3.5)	5.1 (4.3–6.0)
HR (95% CI)	Reference	1.71 (1.37–2.14)
HR (95% CI)^a^	Reference	1.28 (1.01–1.62)
HR (95% CI)^b^	Reference	1.12 (0.87–1.43)
Sudden cardiovascular death		
*n* (%)	44 (6.3)	48 (11.5)
Event rate per 100 person-years (95% CI)	0.8 (0.6–1.1)	1.6 (1.2–2.2)
HR (95% CI)	Reference	2.02 (1.34–3.05)
HR (95% CI)^a^	Reference	1.55 (1.00–2.42)
HR (95% CI)^b^	Reference	1.49 (0.94–2.34)

AF, atrial fibrillation; CI, confidence interval; HR, hazard ratio.

^a^Adjusted for age, sex, treatment assignment, centre, cardiac resynchronization therapy (preexisting or planned), body mass index, duration of heart failure, New York Heart Association functional class, left ventricular ejection fraction, estimated glomerular filtration rate, history of heart failure hospitalization, hypertension, and diabetes.

^b^Adjusted for the log of N-terminal pro-B-type natriuretic peptide in addition to the variables mentioned above.

Among patients randomized to an ICD, there was no significant difference in the risk of serious device infections between patients with and without AF (3.0% vs. 2.2%, *P* = 0.59). Although the risk of appropriate shock was similar in patients with vs. without AF (11.7% vs. 11.2%, *P=* 0.86), patients with AF were more likely to receive an inappropriate shock than those without AF (8.5% vs. 4.2%, *P* = 0.03).

#### Type of atrial fibrillation at baseline

In unadjusted analyses, both non-permanent and permanent AF were associated with a significantly higher rate of all the outcomes examined (see Supplementary material online, *Table S4*). After adjustment for prognostic variables, these associations were generally attenuated and no longer statistically significant, although there was a trend towards a higher rate of all-cause death and sudden cardiovascular death with non-permanent AF, but not permanent AF.

### Effect of implantable cardioverter-defibrillator implantation on outcomes according to AF status at baseline

#### Any atrial fibrillation at baseline

**Figure 1:**
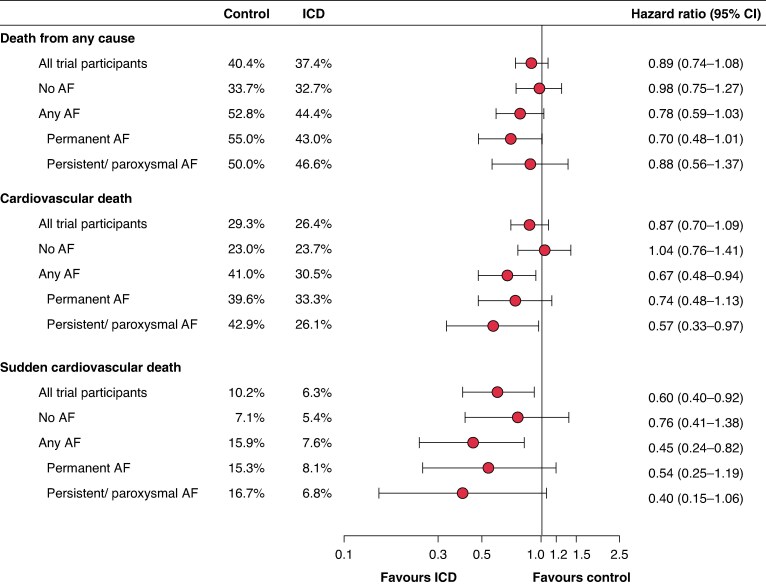
Effect of ICD implantation compared with usual care according to the type of AF status at baseline. AF, atrial fibrillation; CI, confidence interval; HR, hazard ratio; ICD, implantable cardioverter-defibrillator. *All hazard ratios are stratified according to centre and cardiac resynchronization therapy implantation (preexisting or planned).*

The effects of ICD implantation on outcomes according to AF status at baseline are shown in *Table 3* and *Figure 1*. Compared with usual care, ICD implantation did not reduce the rate of all-cause death in patients with [HR 0.78 (95% CI, 0.59–1.03)] or without AF [HR 0.98 (0.75–1.27)], and although AF status did not significantly modify the effect of ICD implantation on this outcome, there was a suggestion of a greater effect in patients with vs. without AF (*P*_interaction_ = 0.15). AF status significantly modified the effect of ICD implantation on cardiovascular death, such that ICD implantation was associated with a lower rate of this outcome in patients with AF [HR 0.67 (0.48–0.94)], but not in those without AF [HR 1.04 (0.76–1.41)] (*P*_interaction_ = 0.04). Although AF status did not significantly modify the effect of ICD implantation on sudden cardiovascular death, there was a suggestion of a greater effect in patients with [HR 0.45 (0.24–0.82)] vs. without AF [HR 0.76 (0.41–1.38)] (*P*_interaction_ = 0.20).

**Table 3:** Effect of ICD implantation compared with usual care according to AF status at baseline

Outcome	No AF		AF		*P*-value for interaction
	*n* = 698		*n* = 418		
	Control group	ICD group	Control group	ICD group	
	*n* = 365	*n* = 333	*n* = 195	*n* = 223	
All-cause death					0.15
*n* (%)	123 (33.7)	109 (32.7)	103 (52.8)	99 (44.4)	
Event rate per 100 person-years (95% CI)	4.4 (3.7–5.2)	4.2 (3.5–5.1)	8.0 (6.6–9.7)	6.1 (5.0–7.4)	
HR (95% CI)^a^	0.98 (0.75–1.27)		0.78 (0.59–1.03)		
Cardiovascular death					0.04
*n* (%)	84 (23.0)	79 (23.7)	80 (41.0)	68 (30.5)	
Event rate per 100 person-years (95% CI)	3.0 (2.4–3.7)	3.0 (2.4–3.8)	6.2 (5.0–7.7)	4.2 (3.3–5.3)	
HR (95% CI)^a^	1.04 (0.76–1.41)		0.67 (0.48–0.94)		
Sudden cardiovascular death					0.20
*n* (%)	26 (7.1)	18 (5.4)	31 (15.9)	17 (7.6)	
Event rate per 100 person-years (95% CI)	0.9 (0.6–1.4)	0.7 (0.4–1.1)	2.4 (1.7–3.4)	1.0 (0.6–1.7)	
HR (95% CI)^a^	0.76 (0.41–1.38)		0.45 (0.24–0.82)		

AF, atrial fibrillation; CI, confidence interval; HR, hazard ratio; ICD, implantable cardioverter-defibrillator.

^a^Stratified according to centre and cardiac resynchronization therapy implantation (preexisting or planned).

**Figure 2:**
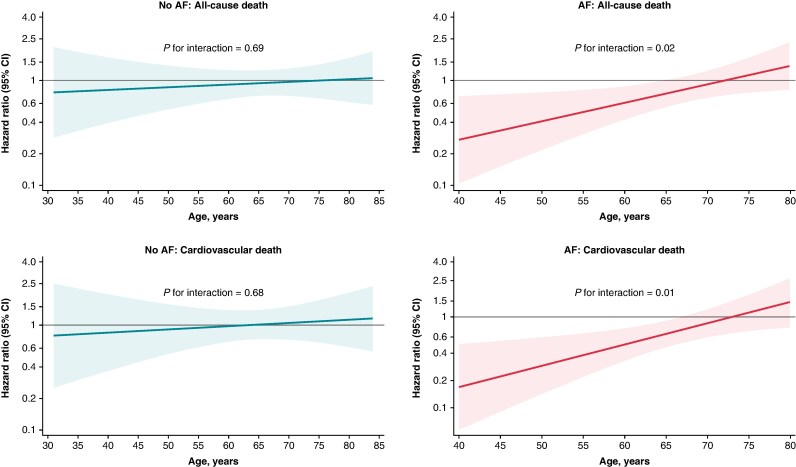
Effect of ICD implantation compared with usual care according to age in patients with and without AF at baseline. The figures show the effect of ICD implantation vs. usual care on death from any cause and cardiovascular death according to continuous age in patients with and without AF. Linear regression models were applied, since these yielded the lowest Akaike information criterion score. All hazard ratios are stratified according to centre and cardiac resynchronization therapy implantation (preexisting or planned). A hazard ratio below 1 favours ICD implantation. AF, atrial fibrillation; CI, confidence interval; ICD, implantable cardioverter-defibrillator.

We also examined the effects of ICD implantation on outcomes according to age in patients with and without AF at baseline. In patients without AF, age did not modify the effect of ICD implantation on all-cause death or cardiovascular death. In patients with AF, age modified the effect of ICD implantation, such that ICD implantation was associated with a lower risk of all-cause death and cardiovascular death among younger, but not older, patients (*Figure 2*).

#### Type of atrial fibrillation at baseline

The effects of ICD implantation on outcomes according to the type of AF at baseline are shown in Supplementary material online, *Table S5* and *Figure 1*. There was no interaction between the type of AF and the effect of ICD implantation on any of the outcomes. However, there was a trend towards a greater benefit of ICD implantation on all outcomes in patients with AF regardless of the type of AF.

## Discussion

In this extended follow-up study of the DANISH trial, AF was common and associated with a higher mortality risk, although this was no longer the case after adjustment for NT-proBNP. There was a trend towards a greater effect of primary prevention ICD implantation, compared with usual clinical care, on all-cause death, cardiovascular death, and sudden cardiovascular death in patients with vs. without AF, although the interaction test was only statistically significant for cardiovascular death.

### Outcomes according to AF status at baseline

It remains uncertain if AF is an independent predictor of adverse outcomes or simply a marker of more advanced HF. The conflicting findings in previous studies may, in part, reflect residual confounding and varying durations of follow-up. In the present analysis of the DANISH trial, during a median follow-up of more than 9 years, AF was associated with a higher mortality risk, including sudden cardiovascular death, even after comprehensive adjustment for potential confounders. However, these associations were no longer significant after adjustment for NT-proBNP, the single strongest predictor of adverse outcomes in HF. These findings are in line with those from a pooled analysis of the Prospective comparison of ARNI with ACEI to Determine Impact on Global Mortality and Morbidity in Heart Failure (PARADIGM-HF) and Aliskiren Trial of Minimizing Outcomes for Patients with Heart Failure (ATMOSPHERE) trials, but not the Dapagliflozin And Prevention of Adverse outcomes in Heart Failure (DAPA-HF) trial, where patients with AF did not have a higher risk of all-cause and cardiovascular death, even in unadjusted analyses.^13,17,20^ Although these observations from the DAPA-HF trial do not support the view that AF is a marker of more advanced HF, the median follow-up of 18 months in the trial might have been too short to detect a mortality difference. Collectively, our observations, coupled with the findings from prior reports, do not fully support the view that AF is an independent prognostic factor of adverse outcomes in patients with HFrEF, but rather a marker of more advanced disease.

### Effect of implantable cardioverter-defibrillator implantation on outcomes according to AF status at baseline

As alluded to in the Introduction, there is some biological plausibility that the effects of primary prevention ICD implantation may differ according to AF status. While the effects of ICD implantation in patients with HFrEF and concomitant AF have been examined in landmark primary prevention ICD trials, the findings have been inconsistent, data on the effects of ICD implantation on sudden cardiac death were not reported, and the proportion of patients with AF was low (≤25%). In the Multicenter Automatic Defibrillator Implantation Trial II (MADIT-II) trial, ICD implantation, compared with usual care, significantly reduced the risk of all-cause death in patients with ischaemic HFrEF, and this beneficial effect was evident in participants with concomitant AF, although a direct comparison of the treatment effect in patients with and without AF was not performed.^10^ Conversely, in the Sudden Cardiac Death in Heart Failure (SCD-HeFT) trial, which enrolled patients with both ischaemic and non-ischaemic HFrEF, the opposite appeared to be true since the beneficial effect of an ICD appeared to be attenuated (or absent) in individuals with AF, although, again, no formal test of interaction was performed.^11^ In the Defibrillators in Non-ischemic Cardiomyopathy Treatment Evaluation (DEFINITE) trial, the association between ICD implantation and all-cause death did not seem to be different in patients with and without AF, although this trial did not demonstrate an overall survival benefit with a primary prevention ICD.^12^

In the DANISH trial, the largest and most contemporary primary prevention ICD trial in patients with non-ischaemic HFrEF, the proportion of patients with AF was substantially higher than in any of the landmark ICD trials (38% vs. ≤25%). In the present extended follow-up analysis of DANISH, AF status significantly modified the effect of ICD implantation on cardiovascular death, such that ICD implantation was associated with a lower rate of this outcome in patients with AF, but not in those without AF. Although the interaction tests for all-cause death and sudden cardiovascular death were not statistically significant, there appeared to be a trend towards a greater effect of ICD implantation on these outcomes in patients with vs. without AF. These effects were generally consistent, irrespective of AF type (i.e. permanent and non-permanent AF).

As with any subgroup analyses, prespecified or not, these cannot provide conclusive evidence of a treatment effect given the lack of statistical power and the risk of spurious associations (i.e. a chance finding). Therefore, the results from the present, and previous, analyses of the effect of ICD implantation according to AF status should be interpreted with caution and considered as hypothesis-generating at best. However, although it is possible that the observed interaction between AF status and the effect of ICD implantation in the DANISH trial may simply have resulted by chance due to multiple testing, there is some biological plausibility for a differential effect of ICD implantation in patients with and without AF. An ICD can prevent sudden cardiovascular death caused by ventricular tachyarrhythmia, severe bradycardia, or complete heart block, but cannot provide protection against other causes of sudden death including ‘pump failure’ or terminal HF. Patients with AF may derive greater benefit from ICD therapy because AF contributes to both ventricular tachyarrhythmias, through mechanisms such as irregular ventricular conduction, short–long–short sequences, structural remodeling, and medication-related pro-arrhythmia, and bradyarrhythmias related to conduction disease or rate-controlling medications. In contrast, in patients without AF, non-arrhythmic mortality may represent a larger competing risk, attenuating the relative benefit of ICD implantation. Indeed, in the DANISH trial, patients with AF had a higher risk of sudden cardiovascular death than those without AF, and the proportion of deaths attributed to sudden cardiovascular death was substantially higher in patients with vs. without AF. Given that the beneficial effect of ICD implantation was mainly observed in younger (and not older) individuals with AF, this hypothesis may be especially relevant in these individuals.

Although our findings support the use of ICD implantation for the prevention of sudden cardiovascular death in patients with non-ischaemic HFrEF and concomitant AF (and especially those of younger age), regardless of AF type, it is important to emphasize that the risk of inappropriate shock is not negligible in these patients. Indeed, despite improvements in ICD devices, the use of less aggressive ICD settings, and the exclusion criterion of dysregulated permanent AF, more than 8% of patients with AF in the DANISH trial experienced inappropriate shocks, corresponding to a two-fold higher risk compared to patients without AF. This highlights the importance of careful patient selection,^21^ optimization of device programming, and continued strategies to reduce the risk of inappropriate shocks in patients with AF.

### Limitations

The main strength of the present study is the long follow-up in the setting of a randomized, controlled multicentre trial with no loss to follow-up. However, there are some important limitations. First, although the assessment of the effect of ICD implantation on death from any cause according to permanent AF status was prespecified, the assessment of the effect of ICD implantation according to any AF status (as well as type of AF), including on secondary outcomes, was not. Second, our findings may not be generalizable to all patients with HFrEF, since the DANISH population was predominantly White. Furthermore, the enrollment of a specific subgroup at higher risk of adverse outcomes such as all-cause mortality and new-onset HF, for example, those with unregulated permanent AF,^22^ was precluded by the prespecified inclusion and exclusion criteria of the DANISH trial. Third, since the DANISH trial only enrolled patients with non-ischaemic HFrEF, our findings cannot be extrapolated to those with HFrEF of ischaemic origin. Fourth, data on the type of AF were investigator-reported, and the possibility of some degree of misclassification cannot be excluded. In addition, data on AF duration at the time of enrollment, new-onset AF or progression of existing AF during follow-up (e.g. from paroxysmal to persistent or permanent AF), treatment of AF during follow-up (including pharmacological treatment and ablation), and echocardiographic data (including data on left atrial size and function) were not available. In this regard, catheter ablation of AF is of particular interest, since it may lead to recovery of left ventricular function in selected patients with non-ischaemic HFrEF, thereby mitigating the indication for ICD implantation and possibly the risk of sudden cardiovascular death.^23–27^ Fifth, the DANISH trial was conducted before angiotensin receptor–neprilysin inhibitors and sodium-glucose cotransporter-2 inhibitors were recommended by HFrEF guidelines, and since both drug classes have been shown to reduce sudden cardiovascular death when added to conventional therapy, the absolute (and relative) risk of sudden cardiovascular death is expected to decline even further, potentially diminishing the benefit of ICD implantation on preventing this mode of death (and perhaps the effect modification of AF observed in this study).^28,29^ Sixth, data on specific subtypes of cardiovascular and sudden cardiovascular death (e.g. ventricular tachyarrhythmia, bradyarrhythmia or pump failure) were not available. Seventh, 56% of the DANISH population received CRT shortly after randomization, with a higher rate in individuals without AF. However, in a previous analysis of the DANISH trial, we demonstrated that the effects of ICD implantation were not modified by CRT.^30^ Finally, given the observational nature of the analyses on the association between AF status and outcomes, the possibility of residual confounding cannot be fully excluded despite adjustment for measured known confounders.

## Conclusions

In this extended follow-up study of DANISH, AF was common and associated with a higher mortality risk, although this was no longer the case after adjustment for NT-proBNP. The presence of AF was associated with a greater effect of ICD implantation on cardiovascular death, and although similar trends were observed for all-cause and sudden cardiovascular death, the treatment-by-subgroup interaction was not statistically significant for these outcomes.

## Supplementary Material

euaf200_Supplementary_Data

## Data Availability

The data that support the findings of this study are available from the corresponding author on reasonable request.
