# Supplementary material for: Atrial fibrillation and implantable cardioverter-defibrillator in non-ischaemic heart failure with reduced ejection fraction: insights from the DANISH trial
Source: Europace. 2025 Sep 24;27(9):euaf200. doi: 10.1093/europace/euaf200 (PMC12457820; doi:10.1093/europace/euaf200)

**SUPPLEMENTAL MATERIAL**

**Atrial Fibrillation and Implantable Cardioverter-Defibrillator in Non-ischemic Heart Failure with Reduced Ejection Fraction: Insights from the DANISH trial**

Seiko N. Doi, MD, PhD; Adelina Yafasova, MD; Jens Jakob Thune, MD, PhD;

Jens C. Nielsen, MD, DMSc; Niels E. Bruun, MD, DMSc; Lars Videbæk, MD, PhD;

Hans Eiskjær, MD, DMSc; Christian Hassager, MD, DMSc;

Jesper H. Svendsen, MD, DMSc; Dan E. Høfsten, MD, PhD; Steen Pehrson, MD, DMSc;

Lars Køber, MD, DMSc; Jawad H. Butt, MD, PhD

**Supplementary Table 1.** **Baseline characteristics of the study population according to treatment assignment and AF at baseline**

|  | No AF  N=698 | | | AF  N=418 | | |
| --- | --- | --- | --- | --- | --- | --- |
|  | Control group  N=365 | ICD group  N=333 | P-value | Control group  N=195 | ICD group  N=223 | P-value |
| Age, median (interquartile range) | 61 (55-68) | 62 (54-71) | 0.16 | 66 (60-72) | 66 (60-72) | 0.97 |
| Male sex, N (%) | 245 (67.1) | 216 (64.9) | 0.53 | 159 (81.5) | 189 (84.8) | 0.38 |
| Physiologic measures, median (interquartile range) |  |  |  |  |  |  |
| Systolic blood pressure, mmHg | 125 (111-139) | 124 (110-141) | 0.70 | 122 (111-136) | 120 (109-136) | 0.46 |
| Heart rate, bpm | 68 (62-77) | 68 (60-76) | 0.53 | 70 (60-80) | 69 (61-77) | 0.59 |
| BMI, kg/m^2^ | 26 (23-30) | 26 (24-30) | 0.87 | 27 (25-31) | 28 (25-32) | 0.59 |
| NT-proBNP, pg/mL | 943 (477-1,931) | 1,034 (511-2,142) | 0.24 | 1,468 (712-2,817) | 1,541 (820-2,591) | 0.64 |
| eGFR, mL/min/1.73cm^2^ | 77 (61-96) | 78 (63-96) | 0.69 | 66 (55-82) | 65 (49-82) | 0.27 |
| QRS duration, msec | 148 (116-164) | 150 (119-169) | 0.16 | 138 (101-164) | 136 (108-162) | 0.87 |
| LVEF, %, mean (SD) | 23.8 (6.4) | 23.7 (6.5) | 0.88 | 24.6 (5.6) | 24.7 (6.1) | 0.89 |
| Duration of HF, median (interquartile range), months | 13 (7-48) | 13 (7-60) | 0.91 | 28 (12-90) | 38 (12-86) | 0.33 |
| Main cause of HF, N (%) |  |  | 0.92 |  |  | 0.26 |
| Idiopathic | 277 (75.9) | 252 (75.7) |  | 148 (75.9) | 172 (77.1) |  |
| Valvular | 9 (2.5) | 11 (3.3) |  | 12 (6.2) | 9 (4.0) |  |
| Hypertension | 42 (11.5) | 38 (11.4) |  | 13 (6.7) | 24 (10.8) |  |
| Other | 37 (10.1) | 32 (9.6) |  | 22 (11.3) | 18 (8.1) |  |
| NYHA class, N (%) |  |  | 0.98 |  |  | 0.94 |
| II | 201 (55.1) | 183 (55.0) |  | 99 (50.8) | 114 (51.1) |  |
| III/IV | 164 (44.9) | 150 (45.1) |  | 96 (49.2) | 109 (48.9) |  |
| Medical history, N (%) |  |  |  |  |  |  |
| Hospitalization for HF | 229 (63.3) | 203 (61.1) | 0.57 | 133 (68.9) | 156 (70.3) | 0.76 |
| Hypertension | 108 (29.6) | 101 (30.3) | 0.83 | 59 (30.4) | 80 (35.9) | 0.24 |
| Diabetes | 73 (20.0) | 54 (16.2) | 0.20 | 39 (20.0) | 45 (20.2) | 0.96 |
| Stroke | 32 (8.8) | 26 (7.8) | 0.65 | 32 (16.5) | 26 (11.7) | 0.16 |
| COPD | 50 (13.8) | 32 (9.6) | 0.09 | 30 (15.8) | 23 (10.5) | 0.11 |
| Treatment, N (%) |  |  |  |  |  |  |
| ACEI/ARB | 354 (97.0) | 321 (96.4) | 0.66 | 190 (97.4) | 212 (95.1) | 0.21 |
| Beta-blocker | 340 (93.2) | 303 (91.0) | 0.29 | 177 (90.8) | 206 (92.4) | 0.55 |
| MRA | 214 (58.6) | 197 (59.2) | 0.89 | 106 (54.4) | 129 (57.9) | 0.47 |
| Amiodarone | 8 (2.2) | 4 (1.2) | 0.31 | 24 (12.3) | 30 (13.5) | 0.73 |
| Loop diuretic | 263 (72.1) | 235 (70.6) | 0.66 | 155 (79.5) | 180 (80.7) | 0.75 |
| Antiplatelet | 175 (48.0) | 165 (49.6) | 0.67 | 58 (29.7) | 63 (28.3) | 0.74 |
| Anticoagulant | 45 (12.3) | 44 (13.2) | 0.73 | 156 (80.0) | 181 (81.2) | 0.76 |
| Preexisting or planned CRT | 223 (61.1) | 207 (62.2) | 0.77 | 100 (51.3) | 115 (51.6) | 0.95 |

*ACEI, angiotensin-converting enzyme inhibitor; AF, atrial fibrillation; ARB, angiotensin receptor blocker; BMI, body mass index; COPD, chronic obstructive pulmonary disease; CRT, cardiac resynchronization therapy; eGFR, estimated glomerular filtration rate; HF, heart failure; LVEF, left ventricular ejection fraction; MRA, mineralocorticoid receptor antagonist; NYHA; New York Heart Association; NT-proBNP, N-terminal pro-B-type natriuretic peptide.*

**Supplementary Table 2. Baseline characteristics of the study population according to type of AF at baseline**

|  | No AF  N=698 | Non-permanent AF  N=172 | Permanent AF  N=246 | P-value |
| --- | --- | --- | --- | --- |
| Age, median (interquartile range) | 62 (54-69) | 65 (58-71) | 67 (61-73) | <0.001 |
| Male sex, N (%) | 461 (66.1) | 136 (79.1) | 212 (86.2) | <0.001 |
| Physiologic measures, median (interquartile range) |  |  |  |  |
| Systolic blood pressure, mmHg | 125 (110-140) | 120 (110-135) | 123 (110-136) | 0.17 |
| Heart rate, bpm | 68 (61-77) | 68 (60-76) | 70 (61-80) | 0.11 |
| BMI, kg/m^2^ | 26 (23-30) | 28 (25-32) | 28 (25-31) | <0.001 |
| NT-proBNP, pg/mL | 990 (500-2,095) | 1,136 (500-2,438) | 1,745 (1,003-2,764) | <0.001 |
| eGFR, mL/min/1.73cm^2^ | 77 (62-96) | 66 (55-83) | 65 (51-82) | <0.001 |
| QRS duration, msec | 149 (116-166) | 151 (119-170) | 124 (100-157) | <0.001 |
| LVEF, %, mean (SD) | 23.8 (6.4) | 24.6 (5.9) | 24.7 (5.9) | 0.07 |
| Duration of HF, median (interquartile range), months | 13 (7-52) | 29 (11-85) | 37 (12-96) | <0.001 |
| Main cause of HF, N (%) |  |  |  | 0.20 |
| Idiopathic | 529 (75.8) | 131 (76.2) | 189 (76.8) |  |
| Valvular | 20 (2.9) | 10 (5.8) | 11 (4.5) |  |
| Hypertension | 80 (11.5) | 11 (6.4) | 26 (10.6) |  |
| Other | 69 (9.9) | 20 (11.6) | 20 (8.1) |  |
| NYHA class, N (%) |  |  |  | 0.42 |
| II | 384 (55.0) | 88 (51.2) | 125 (50.8) |  |
| III/IV | 314 (45.0) | 84 (48.8) | 121 (49.2) |  |
| Medical history, N (%) |  |  |  |  |
| Hospitalization for HF | 432 (62.3) | 126 (73.7) | 163 (66.8) | 0.02 |
| Hypertension | 209 (29.9) | 48 (27.9) | 91 (37.1) | 0.07 |
| Diabetes | 127 (18.2) | 37 (21.5) | 47 (19.1) | 0.61 |
| Stroke | 58 (8.3) | 19 (11.2) | 39 (15.9) | 0.004 |
| COPD | 82 (11.8) | 22 (13.0) | 31 (12.9) | 0.85 |
| Treatment, N (%) |  |  |  |  |
| ACEI/ARB | 675 (96.7) | 164 (95.4) | 238 (96.8) | 0.67 |
| Beta-blocker | 643 (92.1) | 156 (90.7) | 227 (92.3) | 0.81 |
| MRA | 411 (58.9) | 96 (55.8) | 139 (56.5) | 0.68 |
| Amiodarone | 12 (1.7) | 39 (22.7) | 15 (6.1) | <0.001 |
| Loop diuretic | 498 (71.4) | 136 (79.1) | 199 (80.9) | 0.004 |
| Antiplatelet | 340 (48.7) | 61 (35.5) | 60 (24.4) | <0.001 |
| Anticoagulant | 89 (12.8) | 118 (68.6) | 219 (89.0) | <0.001 |
| Preexisting or planned CRT | 430 (61.6) | 107 (62.2) | 108 (43.9) | <0.001 |

*ACEI, angiotensin-converting enzyme inhibitor; AF, atrial fibrillation; ARB, angiotensin receptor blocker; BMI, body mass index; COPD, chronic obstructive pulmonary disease; CRT, cardiac resynchronization therapy; eGFR, estimated glomerular filtration rate; HF, heart failure; LVEF, left ventricular ejection fraction; MRA, mineralocorticoid receptor antagonist; NYHA; New York Heart Association; NT-proBNP, N-terminal pro-B-type natriuretic peptide.*

**Supplementary Table 3.** **Baseline characteristics of the study population according to treatment assignment and type of AF at baseline**

|  | No AF  N=698 | | | Non-permanent AF  N=172 | | | Permanent AF  N=246 | | |
| --- | --- | --- | --- | --- | --- | --- | --- | --- | --- |
|  | Control group  N=365 | ICD group  N=333 | P-value | Control group  N=84 | ICD group  N=88 | P-value | Control group  N=111 | ICD group  N=135 | P-value |
| Age, median (interquartile range) | 61 (55-68) | 62 (54-71) | 0.16 | 65 (58-74) | 65 (59-71) | 0.76 | 67 (61-72) | 67 (61-73) | 0.63 |
| Male sex, N (%) | 245 (67.1) | 216 (64.9) | 0.53 | 61 (72.6) | 75 (85.2) | 0.04 | 98 (88.3) | 114 (84.4) | 0.38 |
| Physiologic measures, median (interquartile range) |  |  |  |  |  |  |  |  |  |
| Systolic blood pressure, mmHg | 125 (111-139) | 124 (110-141) | 0.70 | 112 (110-135) | 125 (110-135) | 0.49 | 124 (113-137) | 119 (107-136) | 0.11 |
| Heart rate, bpm | 68 (62-77) | 68 (60-76) | 0.53 | 69 (59-76) | 68 (61-76) | 0.89 | 72 (61-80) | 69 (61-78) | 0.35 |
| BMI, kg/m^2^ | 26 (23-30) | 26 (24-30) | 0.87 | 27 (24-32) | 28 (26-32) | 0.20 | 28 (25-30) | 28 (25-31) | 0.65 |
| NT-proBNP, pg/mL | 943 (477-1,931) | 1,034 (511-2,142) | 0.24 | 1,092 (479-2,353) | 1,241 (562-2,475) | 0.38 | 1,725 (1,007-2,871) | 1,761 (989-2,622) | 0.79 |
| eGFR, mL/min/1.73cm^2^ | 77 (61-96) | 78 (63-96) | 0.69 | 68 (56-85) | 63 (50-82) | 0.26 | 65 (55-80) | 65 (49-84) | 0.68 |
| QRS duration, msec | 148 (116-164) | 150 (119-169) | 0.16 | 152 (116-174) | 150 (120-166) | 0.74 | 120 (96-160) | 126 (104-153) | 0.46 |
| LVEF, %, mean (SD) | 23.8 (6.4) | 23.7 (6.5) | 0.88 | 24.9 (5.7) | 24.3 (6.1) | 0.52 | 24.4 (5.5) | 24.9 (6.1) | 0.47 |
| Duration of HF, median (interquartile range), months | 13 (7-48) | 13 (7-60) | 0.91 | 22 (10-90) | 37 (11-84) | 0.47 | 36 (12-93) | 38 (14-96) | 0.59 |
| Main cause of HF, N (%) |  |  | 0.92 |  |  | 0.11 |  |  | 0.46 |
| Idiopathic | 277 (75.9) | 252 (75.7) |  | 63 (75.0) | 68 (77.3) |  | 85 (76.6) | 104 (77.0) |  |
| Valvular | 9 (2.5) | 11 (3.3) |  | 8 (9.5) | 2 (2.3) |  | 4 (3.6) | 7 (5.2) |  |
| Hypertension | 42 (11.5) | 38 (11.4) |  | 3 (3.6) | 8 (9.1) |  | 10 (9.0) | 16 (11.9) |  |
| Other | 37 (10.1) | 32 (9.6) |  | 10 (11.9) | 10 (11.4) |  | 12 (10.8) | 8 (5.9) |  |
| NYHA class, N (%) |  |  | 0.98 |  |  | 0.36 |  |  | 0.38 |
| II | 201 (55.1) | 183 (55.0) |  | 46 (54.8) | 42 (47.7) |  | 53 (47.8) | 72 (53.3) |  |
| III/IV | 164 (44.9) | 150 (45.1) |  | 38 (45.2) | 46 (52.3) |  | 58 (52.3) | 63 (46.7) |  |
| Medical history, N (%) |  |  |  |  |  |  |  |  |  |
| Hospitalization for HF | 229 (63.3) | 203 (61.1) | 0.57 | 62 (74.7) | 64 (72.7) | 0.77 | 71 (64.6) | 92 (68.7) | 0.50 |
| Hypertension | 108 (29.6) | 101 (30.3) | 0.83 | 21 (25.0) | 27 (30.7) | 0.41 | 38 (34.6) | 53 (39.3) | 0.45 |
| Diabetes | 73 (20.0) | 54 (16.2) | 0.20 | 19 (22.6) | 18 (20.5) | 0.73 | 20 (18.0) | 27 (20.0) | 0.69 |
| Stroke | 32 (8.8) | 26 (7.8) | 0.65 | 11 (13.3) | 8 (9.2) | 0.40 | 21 (18.9) | 18 (13.3) | 0.23 |
| COPD | 50 (13.8) | 32 (9.6) | 0.09 | 12 (14.6) | 10 (11.5) | 0.54 | 18 (16.7) | 13 (9.9) | 0.12 |
| Treatment, N (%) |  |  |  |  |  |  |  |  |  |
| ACEI/ARB | 354 (97.0) | 321 (96.4) | 0.66 | 82 (97.6) | 82 (93.2) | 0.17 | 108 (97.3) | 130 (96.3) | 0.66 |
| Beta-blocker | 340 (93.2) | 303 (91.0) | 0.29 | 75 (89.3) | 81 (92.1) | 0.53 | 102 (91.9) | 125 (92.6) | 0.84 |
| MRA | 214 (58.6) | 197 (59.2) | 0.89 | 47 (56.0) | 49 (55.7) | 0.97 | 59 (53.2) | 80 (59.3) | 0.34 |
| Amiodarone | 8 (2.2) | 4 (1.2) | 0.31 | 15 (17.9) | 24 (27.3) | 0.14 | 9 (8.1) | 6 (4.4) | 0.23 |
| Loop diuretic | 263 (72.1) | 235 (70.6) | 0.66 | 65 (77.4) | 71 (80.7) | 0.59 | 90 (81.2) | 109 (80.7) | 0.95 |
| Antiplatelet | 175 (48.0) | 165 (49.6) | 0.67 | 26 (31.0) | 35 (39.8) | 0.23 | 32 (28.8) | 28 (20.7) | 0.14 |
| Anticoagulant | 45 (12.3) | 44 (13.2) | 0.73 | 58 (69.1) | 60 (68.2) | 0.90 | 98 (88.3) | 121 (89.6) | 0.74 |
| Preexisting or planned CRT | 223 (61.1) | 207 (62.2) | 0.77 | 51 (60.7) | 56 (63.6) | 0.69 | 49 (44.1) | 59 (43.7) | 0.94 |

*ACEI, angiotensin-converting enzyme inhibitor; AF, atrial fibrillation; ARB, angiotensin receptor blocker; BMI, body mass index; COPD, chronic obstructive pulmonary disease; CRT, cardiac resynchronization therapy; eGFR, estimated glomerular filtration rate; HF, heart failure; LVEF, left ventricular ejection fraction; MRA, mineralocorticoid receptor antagonist; NYHA; New York Heart Association; NT-proBNP, N-terminal pro-B-type natriuretic peptide.*

**Supplementary Table 4. Outcomes according to the type of AF status at baseline**

|  | No AF  N=698 | Non-permanent AF  N=172 | Permanent AF  N=246 |
| --- | --- | --- | --- |
| All-cause death |  |  |  |
| N (%) | 232 (33.2) | 83 (48.3) | 119 (48.4) |
| Event rate per 100 person-years (95% CI) | 4.3 (3.8-4.9) | 6.9 (5.6-8.6) | 6.9 (5.8-8.3) |
| HR (95% CI) | Reference | 1.64 (1.27-2.10) | 1.64 (1.32-2.05) |
| HR (95% CI)^*^ | Reference | 1.28 (0.99-1.67) | 1.15 (0.91-1.46) |
| HR (95% CI)^**^ | Reference | 1.19 (0.91-1.55) | 0.98 (0.76-1.25) |
| Cardiovascular death |  |  |  |
| N (%) | 163 (23.4) | 59 (34.3) | 89 (36.2) |
| Event rate per 100 person-years (95% CI) | 3.0 (2.6-3.5) | 4.9 (3.8-6.3) | 5.2 (4.2-6.4) |
| HR (95% CI) | Reference | 1.66 (1.23-2.34) | 1.75 (1.35-2.27) |
| HR (95% CI)^*^ | Reference | 1.28 (0.94-1.75) | 1.28 (0.97-1.69) |
| HR (95% CI)^**^ | Reference | 1.18 (0.86-1.61) | 1.08 (0.81-1.43) |
| Sudden cardiovascular death |  |  |  |
| N (%) | 44 (6.3) | 20 (11.6) | 28 (11.4) |
| Event rate per 100 person-years (95% CI) | 0.8 (0.6-1.1) | 1.7 (1.1-2.6) | 1.6 (1.1-2.4) |
| HR (95% CI) | Reference | 2.05 (1.21-3.48) | 2.01 (1.25-3.22) |
| HR (95% CI)^*^ | Reference | 1.69 (0.97-2.95) | 1.46 (0.87-2.45) |
| HR (95% CI)^**^ | Reference | 1.65 (0.94-2.89) | 1.36 (0.80-2.34) |

*AF, atrial fibrillation; CI, confidence interval; HR, hazard ratio.*

**Adjusted for age, sex, treatment assignment, center, cardiac resynchronization therapy (preexisting or planned), body mass index, duration of heart failure, New York Heart Association functional class, left ventricular ejection fraction, estimated glomerular filtration rate, history of heart failure hospitalization, hypertension, and diabetes.*

***Adjusted for log of N-terminal pro-B-type natriuretic peptide in additional to the variables mentioned above.*

**Supplementary Table 5.** **Effect of ICD implantation compared with usual care according to the type of AF at baseline**

| Outcome | No AF  N=698 | | Non-permanent AF  N=172 | | | Permanent AF  N=246 | | P-value for  Interaction*** | P-value for  Interaction**** |
| --- | --- | --- | --- | --- | --- | --- | --- | --- | --- |
|  | Control group  N=365 | Control group  N=365 | Control group  N=84 | ICD group  N=88 | Control group  N=111 | | ICD group  N=135 |  |  |
| All-cause death | | | | | | | |  |  |
| N (%) | 123 (33.7) | 109 (32.7) | 42 (50.0) | 41 (46.6) | 61 (55.0) | | 58 (43.0) |  |  |
| Event rate per 100 person-years (95% CI) | 4.4 (3.7-5.2) | 4.2 (3.5-5.1) | 7.6 (5.6-10.3) | 6.3 (4.7-8.6) | 8.2 (6.4-10.6) | | 5.9 (4.6-7.7) |  |  |
| HR (95% CI)* | 0.98 (0.75-1.27) | | 0.88 (0.56-1.37) | | | 0.70 (0.48-1.01) | | 0.13 | 0.37 |
| HR (95% CI)** | 0.99 (0.76-1.28) | | 0.86 (0.55-1.36) | | | 0.70 (0.48-1.01) | | 0.13 | 0.39 |
| Cardiovascular death | | | | | | | |  |  |
| N (%) | 84 (23.0) | 79 (23.7) | 36 (42.9) | 23 (26.1) | 44 (39.6) | | 45 (33.3) |  |  |
| Event rate per 100 person-years (95% CI) | 3.0 (2.4-3.7) | 3.0 (2.4-3.8) | 6.5 (4.7-9.0) | 3.5 (2.4-5.3) | 5.9 (4.4-8.0) | | 4.6 (3.4-6.2) |  |  |
| HR (95% CI)* | 1.04 (0.76-1.41) | | 0.57 (0.33-0.97) | | | 0.74 (0.48-1.13) | | 0.13 | 0.49 |
| HR (95% CI)** | 1.04 (0.77-1.42) | | 0.56 (0.32-0.97) | | | 0.74 (0.48-1.13) | | 0.13 | 0.49 |
| Sudden cardiovascular death | | | | | | | |  |  |
| N (%) | 26 (7.1) | 18 (5.4) | 14 (16.7) | 6 (6.8) | 17 (15.3) | | 11 (8.1) |  |  |
| Event rate per 100 person-years (95% CI) | 0.9 (0.6-1.4) | 0.7 (0.4-1.1) | 2.5 (1.5-4.3) | 0.9 (0.4-2.1) | 2.3 (1.4-3.7) | | 1.1 (0.6-2.0) |  |  |
| HR (95% CI)* | 0.76 (0.41-1.38) | | 0.40 (0.15-1.06) | | | 0.54 (0.25-1.19) | | 0.35 | 0.60 |
| HR (95% CI)** | 0.76 (0.41-1.39) | | 0.41 (0.15-1.09) | | | 0.54 (0.25-1.19) | | 0.36 | 0.60 |

*AF, atrial fibrillation; CI, confidence interval; HR, hazard ratio; ICD, implantable cardioverter-defibrillator.*

**Stratified according to center and cardiac resynchronization therapy implantation (preexisting or planned).*

***Stratified according to center and cardiac resynchronization therapy implantation (preexisting or planned) and adjusted for sex.*

****Interaction between AF type – including the no AF group – and the effect of implantable cardioverter-defibrillator therapy.*

*****Interaction between AF type – excluding the no AF group – and the effect of implantable cardioverter-defibrillator therapy.*

**Supplementary Figure 1. Effect of ICD implantation compared with usual clinical care according to AF at baseline**

*a) Death from any cause*


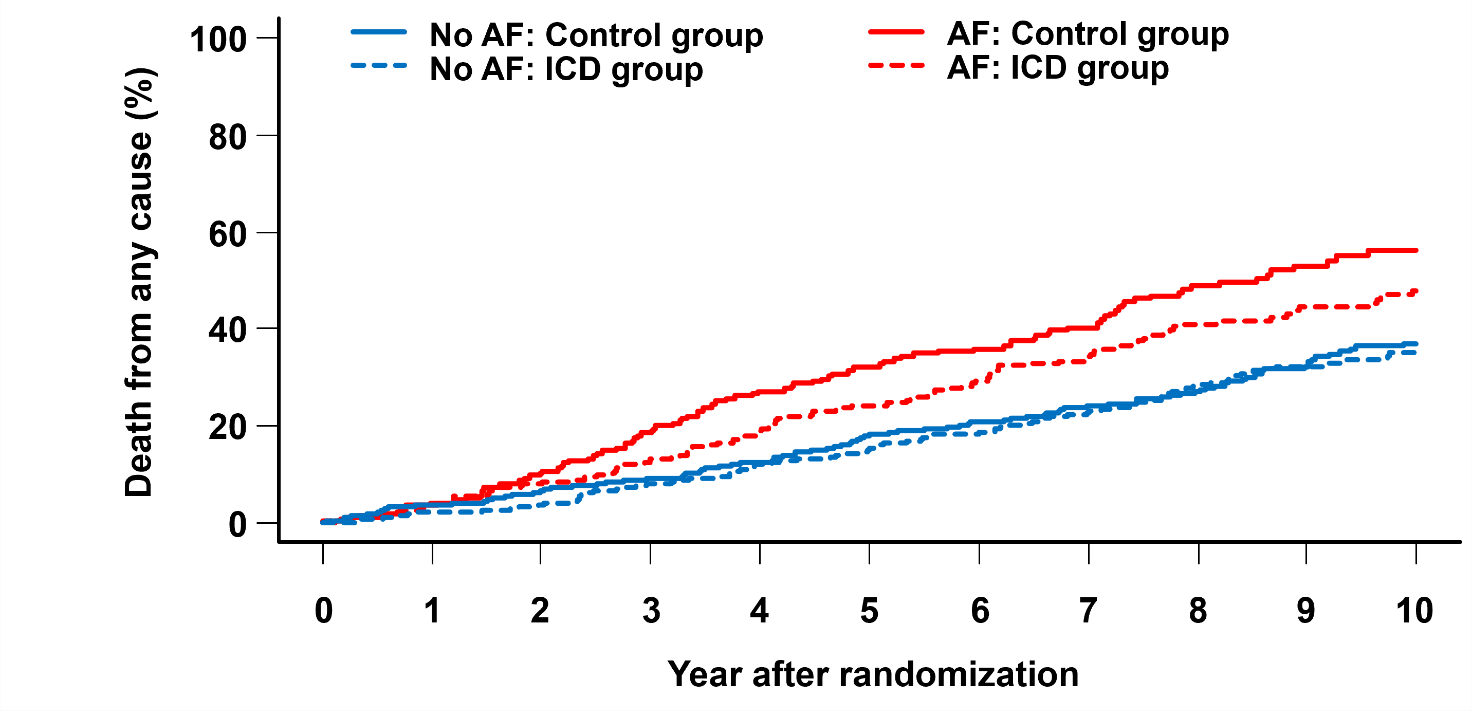


*b) Cardiovascular death*


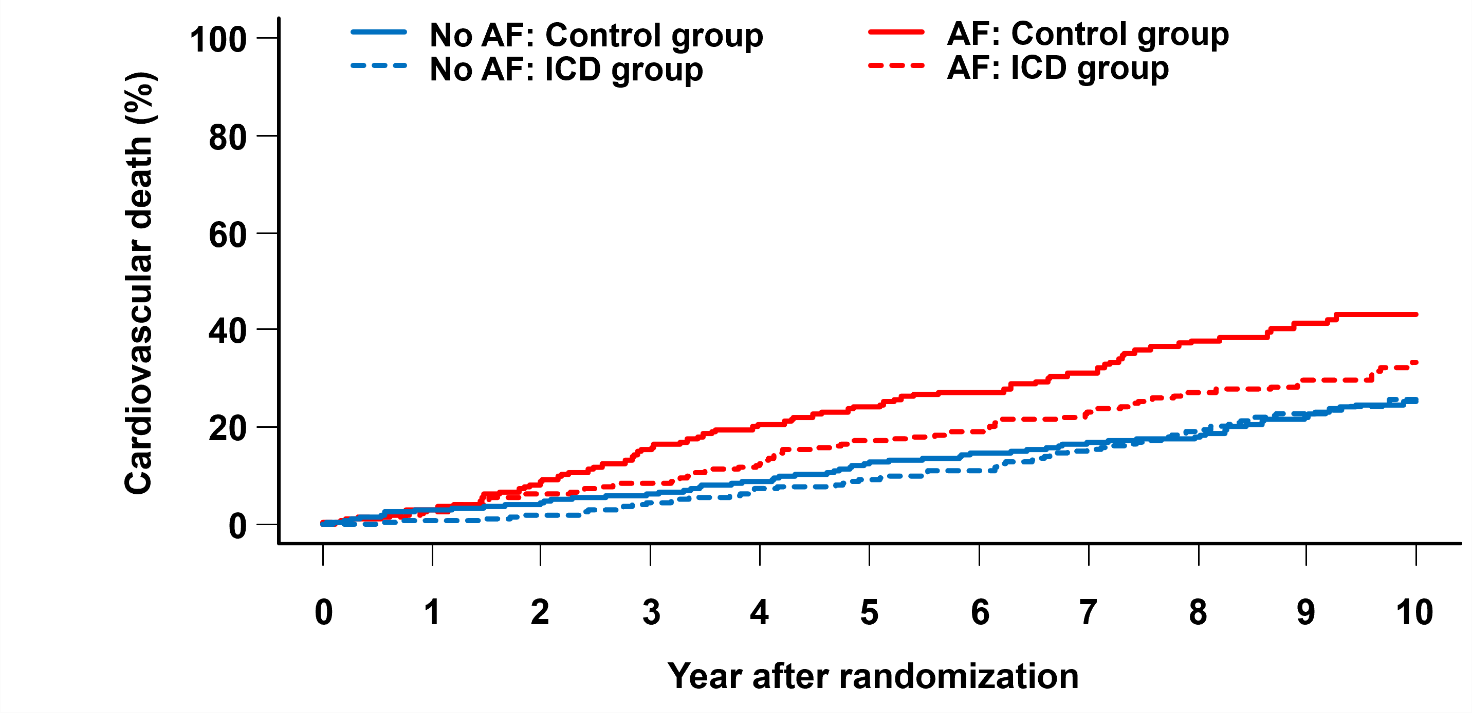


*c) Sudden cardiovascular death*


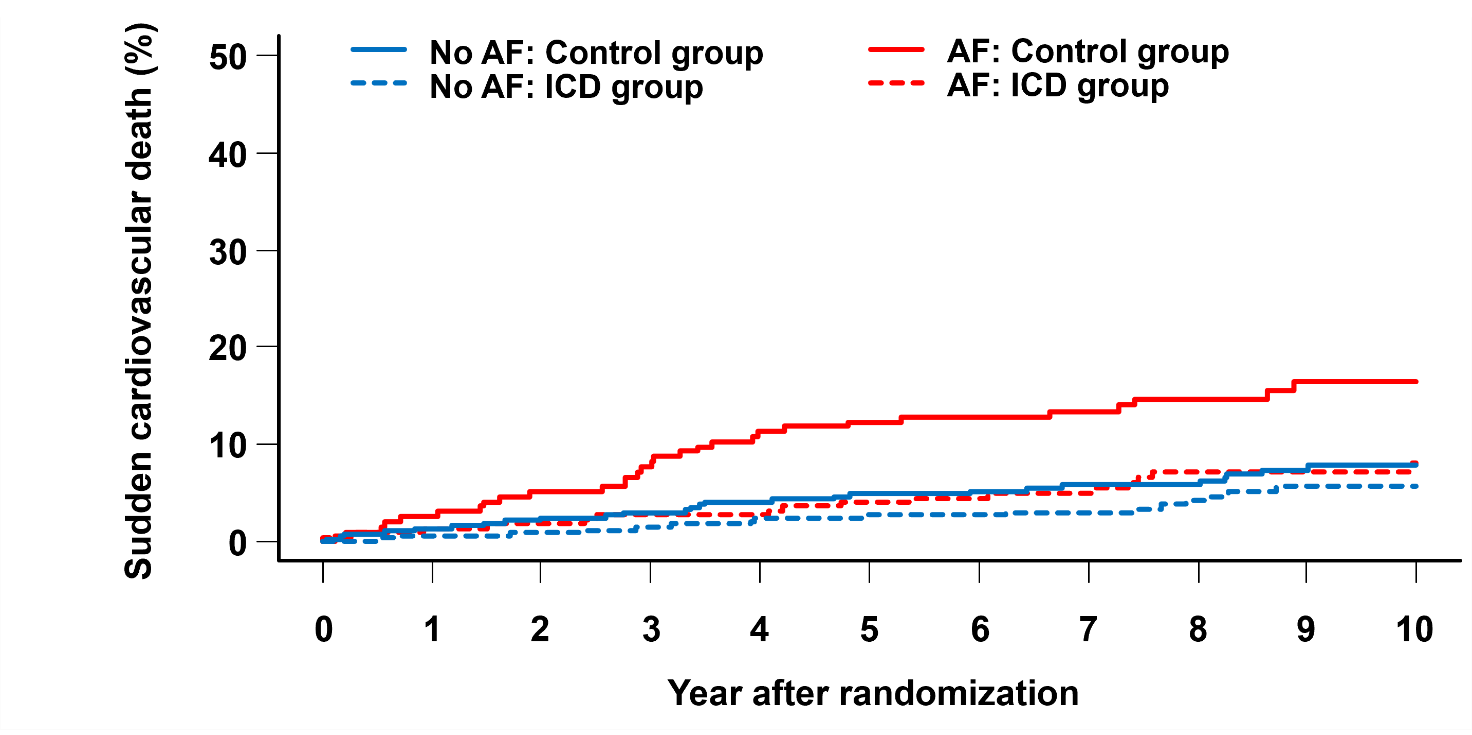

Supplement: euaf200_Supplementary_Data [file euaf200_supplementary_data.docx]
